# Adapting *Connect for Health* pediatric weight management program for telehealth in response to the COVID-19 pandemic

**DOI:** 10.1186/s43058-023-00523-2

**Published:** 2023-11-16

**Authors:** Meg Simione, Kelly Aschbrenner, Haley Farrar-Muir, Man Luo, Jazmin Granadeno, Ariadne Caballero-Gonzalez, Sarah N. Price, Carlos Torres, Alexy Arauz Boudreau, Lauren Fiechtner, Simon J. Hambidge, Kerry Sease, Elsie M. Taveras

**Affiliations:** 1 Division of General Academic Pediatrics, Department of Pediatrics, Mass General for Children, 125 Nashua St, Suite 860, Boston, MA 02114 USA; 2 Department of Pediatrics, Harvard Medical School, Boston, MA USA; 3 Department of Psychiatry, Geisel School of Medicine at Dartmouth, Lebanon, NH USA; 4 MGH Chelsea HealthCare Center, Chelsea, MA USA; 5 Ambulatory Care Services, Denver Health, Denver, CO USA; 6 Department of Pediatrics, University of Colorado School of Medicine, Aurora, CO USA; 7 Department of Pediatrics, University of South Carolina School of Medicine, Greenville, SC USA; 8 Prisma Health, Greenville, SC USA

**Keywords:** Childhood obesity, Telehealth, Implementation science, Adaptations, Limited English proficiency, Equity

## Abstract

**Background:**

To address the evolving needs and context changes due to the COVID-19 pandemic, we adapted *Connect for Health*, an evidence-based, primary care, pediatric weight management intervention. The objective of this study is to describe the planned adaptation process to ensure continued and equitable program uptake during the pandemic.

**Methods:**

Guided by adaptation frameworks, we identified the core functions and forms of *Connect for Health* and then adapted the intervention in response to a changing healthcare context. We engaged stakeholders and surveyed parents of children with a BMI ≥ 85th percentile and pediatric clinicians and examined their experiences using telehealth for pediatric weight management and needs and preferences. Using multivariable logistic regression, we examined the preferences of parents with limited English proficiency regarding key aspects of pediatric weight management.

**Results:**

We surveyed 200 parents and 43% had a primary language of Spanish. Parents wanted care to be a combination of in-person and virtual visits (80%). We found that parents with limited English proficiency had a higher odds ratio of affirming in-person visits are better than virtual visits for ensuring their child’s health concern can be taken care of (OR: 2.91; 95% CI: 1.36, 6.21), feeling comfortable when discussing personal information (OR: 3.91; 95% CI: 1.82, 8.43), talking about healthy behaviors and setting goals (OR: 3.09; 95% CI: 1.39, 6.90), and talking about mental health and overall well-being (OR: 4.02; 95% CI: 1.83, 8.87) than parents without limited English proficiency. We surveyed 75 clinicians and 60% felt telehealth was a useful tool to provide care for pediatric weight management. Clinicians felt virtual visits did not pose barriers to all aspects of care. Informed by the surveys and stakeholder input, we made clinician- and family-level adaptations while retaining the program’s function.

**Conclusions:**

By engaging stakeholders and adapting the program for telehealth, we optimized the reach and fit of *Connect for Health* to ensure its continued uptake. We have provided a real-world example of how clinical innovations can evolve and how to systematically plan adaptations in response to changing healthcare contexts.

**Trial registration:**

Clinicaltrials.gov (NCT04042493), Registered on August 2, 2019.

**Supplementary Information:**

The online version contains supplementary material available at 10.1186/s43058-023-00523-2.

Contributions to the literature

- The COVID-19 pandemic has dramatically affected pediatric primary care resulting in the need to adapt an evidence-based pediatric weight management program for telehealth during implementation.
- Using adaptation frameworks, we engaged clinician and parent stakeholders to examine their experiences, needs, and preferences with telehealth in response to a changing healthcare context during the pandemic. We further examined the preferences of parents with limited English proficiency to ensure the adaptations made were equitable.
- This study provides a real-world example of how an evidence-based intervention can be adapted during implementation to suit changing healthcare contexts and provides direction for systematically planning and making the adaptations.

## Introduction

The COVID-19 pandemic has dramatically affected pediatric primary care. The intermittent suspension of in-person visits, the increase in use of telehealth, and the overall impact of COVID-19 has created new vulnerabilities for populations that are at risk of poor access to care [1]. For children with obesity, these changes have resulted in missed well-child visits and follow-up care to support weight management and related conditions, thereby exacerbating chronic medical conditions and increasing body mass index (BMI) [2–4]. To address the evolving needs and context changes in pediatric primary care, we adapted *Connect for Health* for telehealth and other impacts of the pandemic and related policies. *Connect for Health* is an evidence-based pediatric weight management program intended for delivery in primary care that leverages clinical and community resources to improve child body mass index (BMI) and family-centered outcomes [5, 6]. The program has family- and clinician-facing tools that required adaptations to ensure continued uptake.

Programs must be flexible and adaptable to respond to changing healthcare contexts to increase the likelihood of sustainability [7], but program developers also need to ensure that adaptations are equitable and reduce barriers to care [8, 9]. Telehealth and other program adaptations may offer many opportunities for patients to access necessary services, but they can also present with inequities and challenges when providing obesity-related care [10, 11]. For example, patient characteristics, such as limited English proficiency (LEP) and socio-economic status, have been identified as barriers to telehealth utilization [12, 13]. During the COVID-19 pandemic, childhood obesity interventions have rapidly transitioned to telehealth and made other necessary program changes. However, there have been limited reports of programs that systematically plan adaptations and engage multi-level stakeholders in this process to make sure modifications meet the needs of clinicians and families [14]. By using established adaptation frameworks from the implementation science literature to guide adaptations and document modifications [15, 16], we can evaluate changes to evidence-based programs to ensure we are providing children with obesity, effective, high-quality care.

The objective of this study is to describe the systematic process we undertook to make real-time adaptations in response to the COVID-19 pandemic to the *Connect for Health* pediatric weight management program to ensure continued and equitable program uptake. Our aims were to (1) systematically describe the planned adaptation process and results of that process and (2) examine the experiences and perceptions of parents and clinicians about using telehealth equitably for pediatric weight management to inform program adaptations. By understanding the experiences and perceptions of parents and clinicians and engaging stakeholders, we can make adaptations to the *Connect for Health* program to ensure it equitably meets the needs of clinicians and families who are at risk of poor access to care.

## Materials and methods

### Connect for Health weight management program

*Connect for Health* is a weight management intervention for children ages 2–12 years for delivery in the primary care setting. The program consists of clinician-facing tools including flagging of children with elevated BMIs, clinical decision support to guide management, and clinician education and training and family-facing tools including educational handouts about behavioral change, social and community-informed text messages, and community resource guides. Implementation strategies to support the uptake of the program are currently being tested; and the program components, implementation strategies, and study protocol have been described in detail previously [6]. The implementation of *Connect for Health* is currently being studied in four healthcare organizations that provide care in low-income communities and have high rates of obesity in Boston, MA, Greenville, SC, and Denver, CO. The study protocol was approved by the Mass General Brigham institutional review board.

### Process for planned adaptations

In the pre-implementation phase, the healthcare organizations participating in the implementation of *Connect for Health* conducted extensive stakeholder engagement [17]. The program was then launched in late 2019, months prior to the nationwide lockdowns due to COVID-19 resulting in disruptions to pediatric primary care. During the early months of the pandemic, the healthcare organizations learned from clinicians and stakeholders that they wanted to continue to use the program due to the rapidly increasing rates of childhood obesity secondary to the pandemic [2, 3], but it was challenging to use the program due to the increased use of telehealth and other changes in pediatric primary care. The research study team made the decision to adapt the program during the implementation phase to align with telehealth usage and other pandemic-related policies and ensure continued equitable access to the program.

The iterative process we undertook to adapt the program for telehealth was guided by the phases of the Planned Adaptation Model that allowed us to balance the tension between retaining the program’s purpose and being responsive to the needs of stakeholders [16]. In the first phase, the program developers identified the core functions (i.e., purpose) and forms (i.e., activities) that would enable us to preserve the program’s purpose as we adapted it [18].

In the second phase, we identified the changing healthcare context that occurred during program implementation due to the pandemic. The purpose of this phase was to understand how pediatric primary care was impacted and the issues that needed to be addressed for continued program uptake and equitable delivery of care. We engaged stakeholders across the healthcare organizations (i.e., organizational level challenges) and conducted parent and clinician surveys (i.e., patient-level) challenges at Massachusetts General Hospital. We first sought feedback from clinical experts in pediatric weight management and telehealth in a series of three working groups from September to November 2020. The purpose of the working groups was to understand current challenges clinicians faced when delivering pediatric weight management care and when using telehealth for weight management and to provide input on the development of the clinician and parent surveys.

The purpose of the parent and clinician surveys was to understand their experiences, perceptions, needs, and preferences when using telehealth for pediatric weight management. In conducting surveys, we also wanted to ensure that the telehealth adaptations would not cause further healthcare inequities for patients who have historically been marginalized by the use of telehealth [10, 11]. We surveyed parents of children with a BMI ≥ 85th percentile who attended primary care or a specialty care weight management program either virtually or in-person from February to September 2021 which was prior to adaptations being made. Survey data was linked with electronic health record (EHR) data for socio-demographics. Surveys were conducted over the phone or via email and were available in English and Spanish. Surveys took approximately 20–30 min to complete and a $25 gift card was provided as compensation. The Research Electronic Data Capture (REDCap, Nashville, TN) was used to collect and manage data. We surveyed pediatric specialty and primary care clinicians who care for children with overweight and obesity from December 2020 to March 2021. Information about the survey was sent via email and participants completed the survey using REDCap. Surveys took approximately 10 min to complete, and $50 was provided as compensation.

Survey questions included constructs that have been previously reported in telehealth utilization studies [19]. Parent questions focused on technical quality, experience, and willingness to use telehealth in the future, and clinician questions focused on the effect on the interaction, usefulness, experience, and resources needed. Questions were developed using the Patient Assessment of Chronic Illness Care [20], the modified-Family Centered Care Assessment Tool [21], The Telehealth Usability Questionnaire [22], and questions used to assess patient experiences of care with telehealth [23]. Several questions were asked of both clinicians and parents to compare responses. We used the Checklist for Reporting of Survey Studies (CROSS) (see Additional file 1).

Following the working groups and conduct of surveys, we then engaged key stakeholders with expertise in telehealth and pediatric weight management and conducted planning sessions with sites implementing *Connect for Health* to review proposed adaptations, discuss feasibility, and generate new ideas. We initially presented them with preliminary findings of the clinician and parent surveys to understand if this was similar to their clinical experiences and a document with suggested adaptations. In meetings, we reviewed the information and sought additional input and ideas.

In the third phase, we adapted the intervention with input from stakeholders and survey results, using the Framework for Reporting Adaptations and Modifications-Enhanced (FRAME) to guide and document the adaptations [15]. Because we were making adaptations in real-time, we used FRAME to ensure adaptations did not impact core program mechanisms and that we made changes at the clinical- and family-level. For example, we assessed each adaptation as to whether it changed a core function of the program. We also initially had more clinician-level modifications and by using FRAME we could focus stakeholder discussions on family-level modifications. The final phase of the Planned Adaptation Model is to evaluate the adapted intervention the results of which will be reported a future publication when the study is complete.

### Statistical analyses

We described clinician and parent characteristics who participated in the survey. Descriptive statistics, including frequency and percentage or mean and standard deviation, were then calculated for the survey questions. We described the participant characteristics and survey descriptive statistics overall and according to language of survey administration and used *χ*^2^ tests for categorical variables and t-tests for continuous variables to compare by language. To further examine the preferences of parents with LEP, we selected four questions that are key to delivering effective pediatric weight management care. The questions asked if a virtual or office visit is better for addressing health concerns, sharing private or personal information, setting goals and talking about healthy behaviors, and talking about mental health and well-being. We used multivariable logistic regression to determine the odds ratio and 95% confidence intervals and adjusted for parent education, visit mode, and child’s age. To test for statistical significance in all analyses, we used a 2-sided alpha level of 0.05. The R Studio Software (version 4.1.0) was used for the statical analyses.

## Results

### Identifying core functions

Following the Planned Adaptation Model phases, we initially identified the core functions and forms of *Connect for Health* (Table 1). This process allowed the research study team to clearly identify the current purpose of the program (core functions) and the activities (core forms) and to make decisions about what adaptations were needed.

**Table 1:** Connect for Health core functions, core forms, and adaptations

**Core functions (purposes)**	**Core forms (activities)**
Identify children ages 2–12 years with elevated BMI during primary care visits	Flag in electronic health record indicating elevated BMI based on height and weight for that day’s visit *Adaptations:* BMI based on height and weight recorded at a visit from previous 3 months
Guide primary care management of children with elevated BMI	Clinical decision support tools in electronic health record containing diagnosis codes, laboratory orders, referrals, and family educational materials *Adaptations:* To assist with efficient workflow, added functionality indicating when an order was last placed
Support parental self-guided family behavior change focusing on sleep; screen time; physical activity; healthy drinks; balanced nutrition plan; stress, bullying and self-care	Family educational, one-page handouts printed during well-child visits or included in the after-visit summary Text-messaging program for parents *Adaptations:* Family educational handouts available to be delivered via patient portal; family behavior change messages developed into brief, family-friendly video; video created to prepare families for in-person and telehealth visits focused on behavior change
Connect families to resources to support behavior change and address social needs	Community resource guide printed or on the website Text-messaging program for parents *Adaptations:* Developed one-page handout about stressful times that included resources pandemic-specific resources delivered via patient portal and website; developed one-page handout with a curated list of websites/social media delivered via patient portal and website
Provide clinician education and training in best practices for pediatric weight management	Virtual learning community for clinicians Clinician training sessions and technical assistance led by clinician champion and practice coach in-person *Adaptations:* Training sessions and technical assistance offered virtually; developed handout for clinicians regarding best practices for virtual pediatric weight management

### Identifying changing healthcare contexts

#### Working groups

Results of the working group with pediatric weight management and telehealth experts revealed difficulty with families accessing telehealth platforms, ensuring families are not lost to follow-up which is essential in pediatric weight management care, and providing healthy lifestyle and community resources to families. The working group also provided direct input into the development of the parent and clinician survey questions and reviewed the final surveys.

#### Parent surveys

**Fig. 1:**
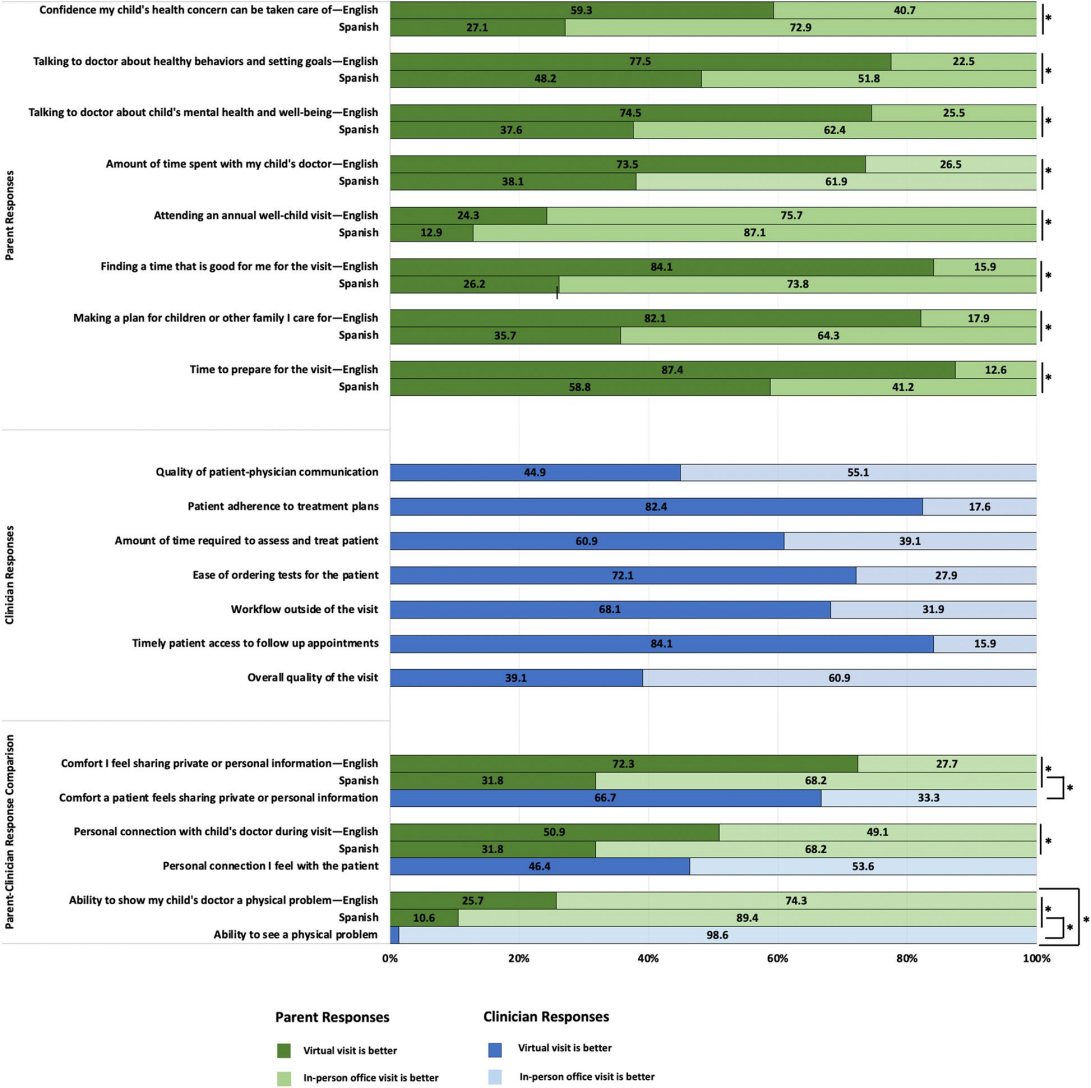
Parent and clinician responses to virtual or in-person visit preferences

We surveyed 200 parents of children with a BMI ≥ 85th percentile and conducted 85 (43%) surveys in Spanish. More parents with LEP were a high school graduate or less (86%) and had an income < $20,000 (46%) than parents without LEP (Table 2). Table 3 describes the results of the parent survey. Fifty percent of participants had a virtual visit (defined as a video or phone visit) for their child and more parents with LEP had the visit conducted via phone (14%) as compared to parents without LEP (7%). Overall, parents did not report any concerns with joining the virtual visit or getting to an in-person visit. When asked about weight management visits, parents reported that they wanted care in the future to be a combination of in-person and virtual visits (80%). Of the parents who attended a virtual visit, a majority reported that the clinician listened to them (89%) and spent enough time with them (88%). Parents without LEP reported they “definitely agreed” that the clinician explained things in an understandable manner (94%) as compared to parents with LEP (74%). When asked if they prefer virtual visits or in-person visits for various reasons (Fig. 1), we found differences between parents with and without LEP. Parents with LEP preferred in-person visits to the virtual visits.

**Table 2:** Characteristics of parents by language (*n* = 200) and clinicians (*n* = 75) who completed the survey

	***n* (%)**			
	**Overall**	**English**	**Spanish**	***p*-value**
**Parent and child characteristics**				
Child’s age mean (SD)	8.15 (2.89)	8.12 (2.94)	8.19 (2.83)	0.87
Childs BMI category				0.79
Overweight (85th–95th %ile)	63 (31.5)	34 (29.6)	29 (34.1)	
Obesity (≥ 95th–99th %ile)	74 (37.0)	44 (38.3)	30 (35.3)	
Severe obesity (≥ 99th %ile)	63 (31.5)	37 (32.2)	26 (30.6)	
Parent age (*n* = 199)				0.004
< 30	19 (9.5)	7 (6.1)	12 (14.1)	
30–39	96 (48.2)	48 (42.1)	48 (56.5)	
40–49	68 (34.2)	45 (39.5)	23 (27.1)	
≥ 50	16 (8.0)	14 (12.3)	2 (2.4)	
Race/ethnicity (*n* = 184)				< 0.001
Hispanic or Latino	117 (63.6)	33 (33.0)	84 (100.0)	
Non-Hispanic White	42 (22.8)	42 (42.0)	0 (0.0)	
Non-Hispanic Black	18 (9.8)	18 (18.0)	0 (0.0)	
Non-Hispanic Asian or Other	7 (3.8)	7 (7.0)	0 (0.0)	
Education (*n* = 195)				< 0.001
High school graduate or less	100 (51.3)	27 (24.5)	73 (85.9)	
More than high school	95 (48.7)	83 (75.5)	12 (14.1)	
Annual Income (*n* = 156)				< 0.001
< $20,000	44 (28.2)	15 (16.1)	29 (46.0)	
$20,001 to $50,000	55 (35.3)	28 (30.1)	27 (42.9)	
** ≥ **$50,000	57 (36.5)	50 (53.8)	7 (11.1)	
**Clinician characteristics**				
Professional role (*n* = 66)				
Physician	59 (89.4)			
Other (ex: psychologist, dietician)	7 (10.6)			
Medical specialty (*n* = 66)				
Primary care	50 (75.8)			
Other (ex: endocrinology, gastroenterology)	16 (24.2)			
Gender (*n* = 66)				
Male	16 (24.2)			
Female	50 (75.8)			
Age (*n* = 65)				
< 40	20 (30.8)			
40–49	16 (24.6)			
50–59	18 (27.7)			
≥ 60	11 (16.9)

**Table 3:** Parent telehealth survey results by language (*n* = 200)

**Visit information**	***n*** **(%)**					***p*-value**
	**Overall**		**English**	**Spanish**		
Reason for visit (*n* = 197)						0.31
Annual well-child visit	88 (44.7)		46 (41.1)	42 (49.4)		
Follow-up appointment	57 (28.9)		34 (30.4)	23 (27.1)		
Weight check	27 (13.7)		14 (12.5)	13 (15.3)		
Other	25 (12.7)		18 (16.1)	7(8.2)		
How was the visit completed?						< 0.001
In-person	101(50.5)		47 (40.9)	54 (63.5)		
Video	79 (39.5)		60 (52.2)	19 (22.4)		
Phone	20 (10.0)		8(7.0)	12 (14.1)		
Reason for virtual visit^a^ (*n* = 98)						0.006
My preference to do a virtual visit	23 (23.5)		20 (29.9)	3 (9.7)		
Due to COVID-19, in-person visits were not allowed	68 (69.4)		40 (59.7)	28 (90.3)		
Unknown	7 (7.1)		7 (10.4)	0 (0.0)		
Utilized interpreter services (*n* = 168)						< 0.001
Yes	58 (34.5)		7 (8.4)	51 (60.0)		
No	110 (65.5)		76 (91.6)	34 (40.0)		
**Visit logistics**						
Was using the video platform easy?^b^ (*n* = 78)						0.31
	Yes, definitely	67 (85.9)	52 (88.1)		15 (78.9)	
	Yes, mostly	8 (10.3)	6 (10.2)		2 (10.5)	
	Yes, somewhat	3 (3.8)	1 (1.7)		2 (10.5)	
Any problems joining the visit?^b^ (*n* = 77)						0.54
	Yes, definitely	3 (3.9)	2 (3.4)		1 (5.3)	
	Yes, somewhat	5 (6.5)	3 (5.2)		2 (10.5)	
	No, I disagree	69 (89.6)	53 (91.4)		16 (84.2)	
Any problems getting to the in-person visit?^c^ (*n* = 96)						0.62
	Yes, definitely	2 (2.1)	0 (0.0)		2 (3.8)	
	Yes, somewhat	7 (7.3)	3 (7.0)		4 (7.5)	
	No, I disagree	87 (90.6)	40 (93.0)		47 (88.7)	
**Virtual visit experience**						
The doctor explained things in a way that was easy to understand^a^ (*n* = 99)						0.01
	Yes, definitely	87 (87.9)	64 (94.1)		23 (74.2)	
	Yes, mostly	9 (9.1)	3 (4.4)		6 (19.4)	
	Yes, somewhat	3 (3.0)	1 (1.5)		2 (6.5)	
The doctor listened carefully to me^a^ (*n* = 98)						0.81
	Yes, definitely	87 (88.8)	60 (89.6)		27 (87.1)	
	Yes, mostly	10 (10.2)	6 (9.0)		4 (12.9)	
	Yes, somewhat	1 (1.0)	1 (1.5)		0 (0.0)	
The doctor spent enough time with me^a^ (*n* = 99)						0.09
	Yes, definitely	87 (87.9)	63 (92.6)		24 (77.4)	
	Yes, mostly	9 (9.1)	4 (5.9)		5 (16.1)	
	Yes, somewhat	3 (3.0)	1 (1.5)		2 (6.5)	
**Preferences**						
In the future, I would want my child’s care to be a combination of in-person and virtual visits (*n* = 196)						0.06
	Agree	157 (80.1)	95 (84.8)		62 (73.8)	
	Disagree	39 (19.9)	17 (15.2)		22 (26.2)	
Virtual visits improve my access to healthcare services (*n* = 192)						0.70
	Agree	142 (74.0)	84 (75.0)		58 (72.5)	
	Disagree	50 (26.0)	28 (25.0)		22 (27.5)	

^a^Respondents who had a video or phone visit were asked the item

^b^Respondents who had a video visit were asked the item

^c^Respondents who had an in-person visit were asked the item

In the multivariable logistic regression models (Table 4), we found parents with LEP had higher odds of affirming that office visits are better than virtual visits for ensuring their child’s health concern can be taken care of (OR: 2.91; 95% CI: 1.36, 6.21), feeling comfortable sharing private or personal information (OR: 3.91; 95% CI: 1.82, 8.43), talking to their child’s doctor about healthy behaviors and setting goals (OR: 3.09; 95% CI: 1.39, 6.90), and talking to their child’s doctor about their child’s mental health and overall well-being (OR: 4.02; 95% CI: 1.83, 8.87) than parents without LEP.

**Table 4:** Associations of telehealth preferences with limited English proficiency status in unadjusted and multivariable adjusted models

	**Unadjusted models**	**Fully adjusted models**^a^
**Outcomes**	**OR (95% CI)**	
Office visits are better than virtual visits for ensuring my child’s health concern can be taken care of during the visit	3.93 (2.14, 7.21)	2.91 (1.36, 6.21)
Office visits are better than virtual visits for feeling comfortable sharing private or personal information	5.61 (3.03, 10.40)	3.91 (1.82, 8.43)
Office visits are better than virtual visits for talking to my child’s doctor about healthy behaviors and setting goals	3.69 (1.99, 6.83)	3.09 (1.39, 6.90)
Office visits are better than virtual visits for talking to my child’s doctor about my child’s mental health and overall well-being	4.85 (2.63, 8.96)	4.02 (1.83, 8.87)

Referent group = parents without limited English proficiency

*OR *Odds ratio, *CI *Confidence interval

^a^Models adjusted for education, visit mode, and child age

#### Clinician surveys

We contacted 94 clinicians and had a response rate of 80%. Of the 75 clinicians surveyed, a majority were physicians (89%) and worked in the primary care setting (76%) (Table 2). More than half of clinicians had experience with virtual visits, had completed more than 50 visits, and had patients on their panels with LEP and limited digital literacy. When examining the effect on interactions (Table 5), clinicians generally felt that virtual visits were not a barrier for ordering labs (72%), providing educational materials (47%), and connecting families to resources (71%). Completing a physical exam (59%) and obtaining anthropometrics (43%) were strong barriers to caring for children with overweight and obesity. Clinicians were equivocal regarding identifying access to the internet, technological issues, privacy and security concerns, and access to medical interpreters as barriers.

**Table 5:** Clinician telehealth survey results (*n* = 75)

**Visit information**	***n*** **(%)**
How many virtual visits have you had in the last 3 months? (*n* = 74)	
None	1 (1.4)
1 to 10	6 (8.1)
11 to 50	22 (29.7)
50 +	45 (60.8)
Proportion of patient panel with limited English proficiency (*n* = 73)	
1–25%	45 (61.6)
25–50%	14 (19.2)
50–75%	7 (9.6)
75–100%	4 (5.5)
Unknown	3 (4.1)
Proportion of patient panel with limited technological/digital literacy (*n* = 74)	
None	2 (2.7)
1–25%	37 (50.0)
25–50%	21 (28.4)
50–75%	8 (10.8)
Unknown	6 (8.1)
**Effect on interaction for virtual visits**	
*Are the following barriers to providing pediatric weight management virtually?*	
Obtaining a height and weight (*n* = 68)	
Strong barrier	29 (42.6)
Somewhat of a barrier	32 (47.1)
Not a barrier	7 (10.3)
Conducting a physical exam (*n* = 66)	
Strong barrier	39 (59.1)
Somewhat of a barrier	23 (34.8)
Not a barrier	4 (6.1)
Ordering labs (*n* = 67)	
Strong barrier	6 (9.0)
Somewhat of a barrier	13 (19.4)
Not a barrier	48 (71.6)
Providing educational materials (*n* = 68)	
Strong barrier	7 (10.3)
Somewhat of a barrier	29 (42.6)
Not a barrier	32 (47.1)
Identifying unmet needs and connecting families to local resources (*n* = 68)	
Strong barrier	2 (2.9)
Somewhat of a barrier	18 (26.5)
Not a barrier	48 (70.6)
Patients’ access to Internet (*n* = 68)	
Strong barrier	19 (27.9)
Somewhat of a barrier	36 (52.9)
Not a barrier	13 (19.1)
Patients’ privacy and security (*n* = 68)	
Strong barrier	3 (4.4)
Somewhat of a barrier	24 (35.3)
Not a barrier	41 (60.3)
Patients’ technical issues to using virtual visit video platforms (*n* = 68)	
Strong barrier	22 (32.4)
Somewhat of a barrier	44 (64.7)
Not a barrier	2 (2.9)
Using medical interpreter services during virtual visits (*n* = 68)	
Strong barrier	21 (30.9)
Somewhat of a barrier	29 (42.6)
Not a barrier	18 (26.5)
**Usefulness of pediatric weight management virtual visits**	
Enough time in a virtual visit to provide necessary care to my patients (*n* = 67)	
Strongly agree	36 (53.7)
Somewhat agree	26 (38.8)
Somewhat disagree	3 (4.5)
Strongly disagree	2 (3.0)
Virtual visits are a valuable tool to enhance pediatric weight management for new patients (*n* = 68)	
Strongly agree	22 (32.4)
Somewhat agree	19 (27.9)
Somewhat disagree	23 (33.8)
Strongly disagree	4 (5.9)
Virtual visits are an effective replacement for follow-up visits with my established patients (*n* = 68)	
Strongly agree	27 (39.7)
Somewhat agree	29 (42.6)
Somewhat disagree	12 (17.6)
Virtual visits would be missed if option was no longer available (*n* = 68)	
Strongly agree	42 (61.8)
Somewhat agree	14 (20.6)
Somewhat disagree	9 (13.2)
Strongly disagree	3 (4.4)
Virtual visits improve a patient’s access to healthcare services (*n* = 68)	
Strongly agree	47 (69.1)
Somewhat agree	19 (27.9)
Somewhat disagree	2 (2.9)
**Resources to improve quality of virtual visits**	
*How helpful are the following resources for pediatric weight management virtual visits?*	
Guidance on best practices for weight management virtual visits (*n* = 67)	
Very helpful	34 (50.7)
Guidance on best practices for weight management virtual visits (Somewhat helpful	31 (46.3)
Not at all helpful/not needed	2 (3.0)
Additional tools in the electronic health record to streamline workflow (*n* = 67)	
Very helpful	20 (29.9)
Somewhat helpful	31 (46.3)
Not at all helpful/not needed	16 (23.9)
Virtual visit resources and tip sheets to provide to patients and families (*n* = 67)	
Very helpful	37 (55.2)
Somewhat helpful	27 (40.3)
Not at all helpful/not needed	3 (4.5)
Guidance on best practices for interpreter services for virtual visits (*n* = 67)	
Very helpful	25 (37.3)
Somewhat helpful	27 (40.3)
Not at all helpful/not needed	15 (22.4)
Improved communication tools to deliver information to families (*n* = 66)	
Very helpful	40 (60.6)
Somewhat helpful	23 (34.8)
Not at all helpful/not needed	3 (4.5)
Resources to address patient accessibility issues (*n* = 67)	
Very helpful	49 (73.1)
Somewhat helpful	16 (23.9)
Not all helpful/not needed	2 (3.0)

Overall, clinicians found telehealth to be useful (Table 5) and strongly agreed or somewhat agreed that virtual visits provided enough time to provide necessary care (93%), enhanced pediatric weight management (60%), were an effective replacement for follow-up patients (82%), and improve patients’ access to healthcare (97%) and that they would miss it if it were no longer an option (82%). Clinicians affirmed the following resources as being useful to enhance virtual visits for pediatric weight management: guidance on best practices for virtual visits, virtual visit resources for families, improved methods for communicating with families, and resources to address patient telehealth accessibility.

Clinicians reported that virtual visits were better for patient adherence to treatment plans, amount of time required for visits, ease of ordering tests, workflow outside of the visit, and timely access to follow-up visits than in-person visits (Fig. 1). Fifty-five percent of clinicians felt that in-person visits were better for patient-clinician communication, and 61% felt the overall quality of in-person visits was better than virtual visits. When comparing parent and clinician responses, we found that parents with LEP felt in-person visits were better for sharing private or personal information (86%) as compared to clinicians (33%).

#### Stakeholder feedback

When we engaged stakeholders and shared preliminary findings of the clinician and parent surveys, we found their clinical experiences to be similar to the findings. We also learned that clinicians wanted multimodal educational resources, virtual resources, and guidance on how to conduct weight management visits virtually. Similar to the survey findings, they also wanted the flexibility to use the program in-person and virtually.

### Adapting the program

Informed by the results of the stakeholder-engaged workgroups and parent surveys, we adapted the content and delivery of *Connect for Health*. We developed a list (using an Excel spreadsheet) that contained the ideas for modifying the program generated throughout the process. The program developers assessed the feasibility and relevance of the potential adaptations, consulted with experts as needed for additional input (for example, consulting with clinical informatics to assess if an electronic health record modification was possible), and made the final list of the planned adaptations (Table 1). During this process, we used FRAME to guide and document the adaptions (Table 6). As intended, the planned adaptations were all to the core forms (versus core functions) of the program, at both the clinician- and family-level to meet the needs of families who are at risk for poor access to care. The adaptations predominately involved refining and adding elements to the program and provided flexibility for the program to be used for in-person or telehealth visits.

**Table 6:** Program adaptations: Framework for Reporting Adaptations and Modifications-Enhanced (FRAME)

**Core function**	**What core forms are modified?**	**At what level of delivery?**	**What is the nature of the content modification?**
Identify children ages 2–12 years with elevated BMI during primary care visits	Content modification: BMI based on height and weight recorded at a visit from previous 3 months	Clinician-level	Tailoring/tweaking/refining
Guide primary care management of children with elevated BMI	Content modification: to assist with efficient workflow, added functionality indicating when an order was last placed	Clinician-level	Tailoring/tweaking/refining
Support parental self-guided family behavior change	Content modification: family educational handouts available to be delivered via patient portal	Family-level	Tailoring/tweaking/refining
Support parental self-guided family behavior change	Content modification: family behavior change messages developed into brief, family-friendly video	Family-level	Adding elements
Support parental self-guided family behavior change	Content modification: video created to prepare families for in-person and telehealth visits focused on behavior change	Family-level	Adding elements
Connect families to resources to support behavior change and address social needs	Content modification: developed one-page handout about stressful times that included pandemic-specific resources delivered via patient portal and website	Family-level	Adding elements
Connect families to resources to support behavior change and address social needs	Content modification: developed one-page handout with a curated list of websites/social media delivered via patient portal and website	Family-level	Adding elements
Provide clinician education and training in best practices for pediatric weight management	Training modification: training sessions and technical assistance offered virtually	Clinician-level	Tailoring/tweaking/refining
Provide clinician education and training in best practices for pediatric weight management	Content modification: developed handout for clinicians regarding best practices for virtual pediatric weight management	Clinician-level	Adding elements

The adaptations addressed telehealth as well as other pandemic-related challenges and allowed us to improve the reach and fit of the program given the changing context of the pandemic. Several of the adaptations were designed to ensure the program met the needs of families who are at risk for poor access to care. We culturally tailored materials regarding behavior change during stressful times, developed video-based educational materials in English and Spanish, provided additional clinician guidance and resources, and optimized the clinical decision support tools and flagging system to integrate into the workflow of virtual visits. The videos reviewed what to expect during a pediatric weight management visit to help familiarize and prepare families. Families could access the video on their mobile phone which based on our previous work was an acceptable method [17], and the brief video length under 2 min would keep data usage at a minimum. The video was created in response to the parent survey findings that in-person visits were better for communicating concerns and talking about healthy behaviors as we felt if families knew what to expect their visit experience would improve. The clinician guidance and resources were developed to address similar parent findings as well as the clinician survey finding that requested virtual weight management guidance to improve visit experience. The electronic health record modifications were in direct response to the clinician survey finding of additional tools to streamline workflow. When making program adaptations, we were mindful of organizational and policy changes that impacted telehealth usage, particularly initiatives to reduce inequities (for example, providing patients options of receiving in-person or virtual care, calling patients before visits to familiarize them with the technology platforms; registering them for patient portal platforms), and aligned clinician and parent guidance accordingly. Our findings from the surveys that families and clinicians wanted options for in-person and telehealth care reinforced organizational policies for the continued use of telehealth after the pandemic ended and made us carefully consider modifications that we made to ensure the flexible use of *Connect for Health* for all visit types.

## Discussion

In this study, we described a systematic process for adapting the *Connect for Health* pediatric weight management program. The results of stakeholder engagement and parent and clinician surveys informed planned adaptations in response to changing healthcare contexts caused by COVID-19. We conducted working group meetings and engaged experts in pediatric weight management and telehealth to inform parent survey development, which in turn informed planned program adaptations. We found that parents wanted their care to be a combination of in-person and virtual visits and clinicians wanted virtual care to remain an option. When we further examined the preferences of parents with LEP, we found that in-person visits were better than virtual visits for ensuring their child’s health concerns can be taken care of, sharing private information, and talking about healthy behaviors and mental health as compared to parents without LEP. Clinicians felt virtual visits posed barriers to some aspects of care and they requested resources to improve virtual visits, particularly for families with limited digital health and English proficiency. Based on the information gathered through stakeholder engagement, our team adapted the program for telehealth and other contextual needs due to the COVID-19 pandemic. We made modifications to the core forms of the program that were tailored to both families and clinicians with special attention to ensure that the adaptations met the needs of families who are at risk of poor access to care.

During the COVID-19 pandemic as compared to a pre-pandemic period, childhood obesity rates have rapidly increased [2, 3], and racial, ethnic, and socio-economic disparities that were preexisting have widened [2]. The alarming rise underscores the importance of primary care management approaches and those approaches remaining adaptable to meet evolving needs. Telehealth is an approach to delivering care to children and their families that can promote equity by improving access and participation in weight management programs [24, 25]. Given the high prevalence of unmet social needs for children with obesity, providing care options that address transportation and time barriers can improve access and increase engagement. Studies prior to and during the pandemic have suggested that telehealth for pediatric weight management is acceptable to families, results in high satisfaction with care, improves show rates, and supports behavior changes [11, 26–29]. We similarly found that families perceived telehealth to be acceptable although we found differences in preferences for virtual or in-person care between parents with and without LEP. In a study conducted before the pandemic and the dramatic rise in telehealth, primary care and specialty care pediatricians were surveyed about their attitudes and experiences with telehealth [30]. Only 13% of clinicians had reported using telehealth compared to almost all of the clinicians in our survey. Sisk and colleagues [30] found that clinicians had identified reasons for not using telehealth including technological barriers, usefulness, and patient reluctance. We found many of these barriers to be of little concern to clinicians presently.

Despite the promise of telehealth improving access to care, telehealth is not without its own barriers to care that need to be addressed [10, 11, 31–33]. Although some barriers need to be addressed at policy levels (i.e., broadband infrastructure and reimbursement), other barriers should be addressed at the programmatic or organizational level. As programs are adapted for telehealth, finding the balance between expanding access to care while honoring the preferences of families will be a challenge. In this study, clinicians and parents affirmed that virtual visits improve access to care and wanted a combination of visits, but parents with LEP felt in-person visits were better for some aspects of care. We reconciled these differences by allowing for flexibility of the program to be delivered virtually or in-person, providing parent education about what to expect during a virtual pediatric weight management visit, and developing clinician virtual guidance to support families. Many of these tools could also be used asynchronously allowing families to engage with materials when it is convenient for them.

We found that parents with LEP reported less of a preference for virtual visits than parents without LEP. Similarly, studies have found that adults with LEP had lower rates of telehealth use and LEP has been identified as an important barrier to use [12, 13]. The barrier might be due to clinicians’ comfort and knowledge using telehealth for non-English speakers and integrating interpreters into visits [34, 35]. Additionally, adults with low incomes, less education, or who are racially and ethnically diverse have been shown to be less willing to use telehealth [36]. In our study, although we only examined differences between LEP status, we did find statistically significant differences between race/ethnicity, income, and education for parents with and without LEP as more parents with LEP were Hispanic/Latino, had an income less than $20,000, and had less education. Consistent with other studies, we also found that parents with LEP were more likely to have had a phone visit rather than a video visit [37–39] indicating that phone visits need to remain an option as solutions to helping families transition to video visits are designed. As we have done in our study, these factors are critical to systematically assess and address given the persistent racial-ethnic and socioeconomic disparities that exist in childhood obesity [11].

The evidence in the literature and our findings are convincing that primary care-based programs should be equitably adapted for virtual care to ensure all children are equally benefitting. Historically, fidelity to the original intervention and a tolerance for the intervention performing worse in other settings than the controlled setting for the trial was accepted [7]. However, the Dynamic Sustainability Framework posits that change should occur in interventions over time in response to the changing context of care and the setting and the broader system should dictate how the intervention is delivered [7]. Without allowing for change, interventions are less likely to produce improved health outcomes and be sustained. We began this process of adaptations during implementation to ensure the program met the changing care context due to the pandemic. We balanced the need for the adaptations while retaining the core functions that were essential to the effectiveness of the program. We surveyed and continually engaged stakeholders to understand their needs and ensure a good fit as well as considered the needs of populations who have been disproportionately affected by health inequities. Baumann and Cabassa [8] state that a critical element of addressing inequities in healthcare is by developing the science of adaptations and to view adaptations as an implementation strategy. This helps to normalize the adaptation process for researchers and clinicians and there is now an emerging literature of implementation science adaptations [40, 41], although few adaptation studies have focused on equity and childhood obesity [42].

Our study is not without limitations. *Connect for Health* is being implemented in four healthcare organizations, but the surveys were only conducted in one setting. To account for this, we iteratively sought feedback from the other organizations and conducted planning sessions with them. These other organizations serve both urban and rural areas, but the surveys were conducted in a region that is predominately urban; therefore, the unique needs of rural areas in regard to telehealth may not all have been addressed. In addition, the surveys helped us to understand the experiences, needs, and preferences and differences between parents with and without LEP. From the surveys, we do not understand the “why” of the differences, and conducting interviews in the future may be beneficial. Given the timeframe of the surveys, we also do not know how attitudes towards telehealth have changed over time.

## Conclusions

Due to COVID-19-related disruptions to in-person care, we adapted *Connect for Health* and documented the process for making planned program adaptations. The results of the parent and clinician surveys and stakeholder engagement informed the adaptations. The adaptations were made to address the changing contexts due to the pandemic and related policies and focused on program flexibility for virtual or in-person visits as offering both modalities will enhance patient care for all. This key finding was pivotal for *Connect for Health* and other programs to ensure equitable access to childhood obesity interventions. The adaptations made were guided by implementation science frameworks and documented to assist with future program evaluation making the process replicable by others to support planned adaptations to meet the evolving and dynamic needs of healthcare settings for continued improvement of health outcomes.

### Supplementary Information

**Additional file 1.** Checklist for Reporting of Survey Studies (CROSS).

## Data Availability

The datasets used during the current study are available from the corresponding author on reasonable request.

## Abbreviations

BMI: Body mass index
LEP: Limited English proficiency
FRAME: Framework for Reporting Adaptations and Modifications-Enhanced
REDCap: Research Electronic Data Capture
OR: Odds ratio

## References

[1] Kamerow D (2020). COVID-19: don’t forget the impact on US family physicians. BMJ.

[2] Jenssen BP, Kelly MK, Powell M, Bouchelle Z, Mayne SL, Fiks AG (2021). COVID-19 and changes in child obesity. Pediatrics..

[3] Lange SJ, Kompaniyets L, Freedman DS, Kraus EM, Porter R, DNP3 (2021). Longitudinal trends in body mass index before and during the COVID-19 pandemic among persons aged 2–19 years - United States, 2018–2020. MMWR Morb Mortal Wkly Rep..

[4] Hu P, Samuels S, Maciejewski KR, Li F, Aloe C, Van Name M, et al. Changes in Weight-Related Health Behaviors and Social Determinants of Health among Youth with Overweight/Obesity during the COVID-19 Pandemic. Child Obes. 2022;18(6):369–82. 10.1089/chi.2021.0196. 10.1089/chi.2021.0196 PMC9492789 34919458

[5] Taveras EM, Marshall R, Sharifi M, Avalon E, Fiechtner L, Horan C (2017). Comparative effectiveness of clinical-community childhood obesity interventions. JAMA Pediatr..

[6] Simione M, Farrar-Muir H, Mini FN, Perkins ME, Luo M, Frost H (2021). Implementation of the Connect for Health pediatric weight management program: study protocol and baseline characteristics. J Comp Eff Res..

[7] Chambers DA, Glasgow RE, Stange KC (2013). The dynamic sustainability framework: addressing the paradox of sustainment amid ongoing change. Implement Sci.

[8] Baumann AA, Cabassa LJ (2020). Reframing implementation science to address inequities in healthcare delivery. BMC Health Serv Res..

[9] Shelton RC, Chambers DA, Glasgow RE (2020). An extension of RE-AIM to enhance sustainability: addressing dynamic context and promoting health equity over time. Front Public Health..

[10] Nouri S, Khoong EC, Lyles CR, Karliner L. Addressing equity in telemedicine for chronic disease management during the COVID-19 pandemic. NEJM Catal Innov Care Deliv. 2020;1(3). https://catalyst.nejm.org/doi/full/10.1056/CAT.20.0123.

[11] Woo Baidal JA, Chang J, Hulse E, Turetsky R, Parkinson K, Rausch JC (2020). Zooming toward a telehealth solution for vulnerable children with obesity during coronavirus disease 2019. Obesity..

[12] Rodriguez JA, Saadi A, Schwamm LH, Bates DW, Samal L (2021). Disparities in telehealth use among California patients with limited English proficiency. Health Aff (Millwood).

[13] Tan-McGrory A, Schwamm LH, Kirwan C, Betancourt JR, Barreto EA (2022). Addressing virtual care disparities for patients with limited English proficiency. Am J Manag Care.

[14] Whitley A, Yahia N (2021). Efficacy of clinic-based telehealth vs. face-to-face interventions for obesity treatment in children and adolescents in the United States and Canada: a systematic review. Childhood Obesity..

[15] Stirman SW, Baumann AA, Miller CJ, Wiltsey Stirman S, Baumann AA, Miller CJ (2019). The FRAME: an expanded framework for reporting adaptations and modifications to evidence-based interventions. Implement Sci.

[16] Lee SJ, Altschul I, Mowbray CT (2008). Using planned adaptation to implement evidence-based programs with new populations. Am J Community Psychol.

[17] Simione M, Frost HM, Cournoyer R, Mini FN, Cassidy J, Craddock C (2020). Engaging stakeholders in the adaptation of the Connect for Health pediatric weight management program for national implementation. Implement Sci Commun..

[18] Kirk MA, Haines ER, Rokoske FS, Powell BJ, Weinberger M, Hanson LC (2021). A case study of a theory-based method for identifying and reporting core functions and forms of evidence-based interventions. Transl Behav Med.

[19] Langbecker D, Caffery LJ, Gillespie N, Smith AC (2017). Using survey methods in telehealth research: a practical guide. J Telemed Telecare.

[20] Glasgow RE, Wagner EH, Schaefer J, Mahoney LD, Reid RJ, Greene SM (2005). Development and validation of the Patient Assessment of Chronic Illness Care (PACIC). Med Care.

[21] Simione M, Sharifi M, Gerber MW, Marshall R, Avalon E, Fiechtner L (2020). Family-centeredness of childhood obesity interventions: psychometrics & outcomes of the family-centered care assessment tool. Health Qual Life Outcomes..

[22] Parmanto B, Lewis AN, Graham KM, Bertolet MH (2016). Development of the Telehealth Usability Questionnaire (TUQ). Int J Telerehabil.

[23] Donelan K, Barreto EA, Sossong S, Michael C, Estrada JJ, Cohen AB (2019). Patient and clinician experiences with telehealth for patient follow-up care. Am J Manag Care.

[24] Dorsey ER, Topol EJ (2016). State of telehealth. N Engl J Med.

[25] Moorman EL, Koskela-Staples NC, Mathai BB, Fedele DA, Janicke DM (2021). Pediatric obesity treatment via telehealth: current evidence and future directions. Curr Obes Rep.

[26] Cueto V, Sanders LM (2020). Telehealth opportunities and challenges for managing pediatric obesity. Pediatr Clin North Am.

[27] DeSilva S, Vaidya SS (2021). The application of telemedicine to pediatric obesity: lessons from the past decade. Telemed E-Health..

[28] Chai LK, Collins CE, May C, Ashman A, Holder C, Brown LJ (2021). Feasibility and efficacy of a web-based family telehealth nutrition intervention to improve child weight status and dietary intake: a pilot randomised controlled trial. J Telemed Telecare.

[29] Eisenburger N, Friesen D, Haas F, Klaudius M, Schmidt L, Vandeven S (2022). Short report: weight management of children and adolescents with obesity during the COVID-19 pandemic in Germany. PLoS ONE.

[30] Sisk B, Alexander J, Bodnar C, Curfman A, Garber K, McSwain SD (2020). Pediatrician attitudes toward and experiences with telehealth sse: results from a national survey. Acad Pediatr..

[31] Scott Kruse C, Karem P, Shifflett K, Vegi L, Ravi K, Brooks M (2018). Evaluating barriers to adopting telemedicine worldwide: a systematic review. J Telemed Telecare.

[32] Knotowicz H, Haas A, Coe S, Furuta GT, Mehta P (2019). Opportunities for innovation and improved care using telehealth for nutritional interventions. Gastroenterology.

[33] Mehta P, Stahl MG, Germone MM, Nagle S, Guigli R, Thomas J (2020). Telehealth and nutrition support during the COVID-19 pandemic. J Acad Nutr Diet..

[34] Shin TM, Ortega P, Hardin K (2021). Educating clinicians to improve telemedicine access for patients with limited English proficiency. Challenges.

[35] Yin L, Ng F, Rutherford-Rojas M, Williams M, Cornes S, Fernandez A (2022). Assessing medical student readiness to navigate language barriers in telehealth: cross-sectional survey study. JMIR Med Educ.

[36] Fischer SH, Ray KN, Mehrotra A, Bloom EL, Uscher-Pines L (2020). Prevalence and characteristics of telehealth utilization in the United States. JAMA Netw Open.

[37] Pierce RP, Stevermer JJ. Disparities in use of telehealth at the onset of the COVID-19 public health emergency. J Telemed Telecare. 2020;1357633X20963893. 10.1177/1357633X20963893 PMC7578842 33081595

[38] Rodriguez JA, Betancourt JR, Sequist TD, Ganguli I (2021). Differences in the use of telephone and video telemedicine visits during the COVID-19 pandemic. Am J Manag Care.

[39] Zachrison KS, Yan Z, Sequist T, Licurse A, Tan-McGrory A, Erskine A, et al. Patient characteristics associated with the successful transition to virtual care: lessons learned from the first million patients. J Telemed Telecare. 2021;1357633X211015547. 10.1177/1357633X211015547 34120506

[40] Escoffery C, Lebow-Skelley E, Haardoerfer R, Boing E, Udelson H, Wood R (2018). A systematic review of adaptations of evidence-based public health interventions globally. Implement Sci.

[41] Rabin BA, McCreight M, Battaglia C, Ayele R, Burke RE, Hess PL (2018). Systematic, multimethod assessment of adaptations across four diverse health systems interventions. Front Public Health.

[42] Aschbrenner KA, Mueller NM, Banerjee S, Bartels SJ. Applying an equity lens to characterizing the process and reasons for an adaptation to an evidenced-based practice. Implement Res Pract. 2. 10.1177/26334895211017252 PMC8428660 34514417

